# Cardio-Respiratory Fitness and Cardiovascular Disease Risk Factors Among South African Medical Students

**DOI:** 10.1177/15598276221089888

**Published:** 2022-05-23

**Authors:** Georgia Torres, Neil F Gordon, Demitri Constantinou

**Affiliations:** Centre for Exercise Science and Sports Medicine, University of the Witwatersrand Faculty of Science, Johannesburg, South Africa GT,NG,DC and INTERVENT International, LLC, Medical and Science Office, Savannah, GA, USA NG and International Federation of Sports Medicine, Lausanne, Switzerland GT,DC

**Keywords:** physical inactivity, aerobic fitness, healthcare providers, heart disease risk

## Abstract

Cardiovascular disease (CVD) risk factors have been associated with CVD mortality, and physicians use CVD risk factor profiles (smoking, dyslipidemia, hypertension, etc.) to address patient health. Furthermore, cardio-respiratory fitness (CRF) has been shown to be an independent risk factor for CVD and all-cause mortality. Cardio-respiratory fitness is also the risk factor that contributes the highest percentage to all-cause deaths when compared to other traditional risk factors. In addition, studies have reported that adding CRF to established CVD risk factors improves the precision of prediction for CVD morbidity and mortality. Medical students tend to adopt sedentary and unhealthy lifestyles during the course of their education that negatively affect CVD risk factors and CRF. The majority of research on CVD risk, health status and lifestyle factors of medical students has used self-reported data and questionnaires for CVD risk factors and not included CRF in the health status measurements. In addition, studies have found that future medical doctors’ own health and lifestyle practices influence their counselling activities. Allowing future medical doctors to assess their personal CVD risk factors and CRF may thus be important in their use of physical activity counselling with patients’ lifestyle management for health benefits and improvement. A descriptive, cross-sectional cohort study design was used with the aim to determine CVD risk factors using CRF measures and physical activity levels in a cohort of South African medical students. The most significant finding was that they were not meeting the PA levels recommended to maintain health and lower CVD risk.

Fifth-year medical students at a South African university were not meeting optimal PA levels and CRF levels were recommended to maintain health and lower CVD risk

## Introduction

Cardiovascular disease (CVD) risk factors have been associated with CVD mortality,^1^ and physicians use CVD risk factor profiles (smoking, dyslipidemia, hypertension, etc.) to address patient health. Furthermore, cardio-respiratory fitness (CRF) has been shown to be an independent risk factor for CVD^2^ and all-cause mortality.^3,4^ Cardio-respiratory fitness is also the risk factor that contributes the highest percentage to all-cause deaths when compared to other traditional risk factors.^5,6^ In addition, studies have reported that adding CRF to established CVD risk factors improves the precision of prediction for CVD morbidity and mortality.^7^

Medical students tend to adopt sedentary and unhealthy lifestyles during the course of their education that negatively affect CVD risk factors and CRF.^8^ The majority of research on CVD risk, health status and lifestyle factors of medical students has used self-reported data and questionnaires for CVD risk factors^9-11^ and not included CRF in the health status measurements.

In addition, studies have found that future medical doctors’ own health and lifestyle practices influence their counselling activities.^12,13^ Allowing future medical doctors to assess their personal CVD risk factors and CRF may thus be important in their use of physical activity (PA) counselling with patients’ lifestyle management for health benefits and improvement.

### Aim

This study aimed to analyse the CVD risk factors, PA and CRF levels measured in a cohort of fifth-year medical students in South Africa (SA), collected as part of teaching and learning activities that aimed to include physical activity in the undergraduate medical curriculum (total 6 years).

### Objectives

The study aimed to describe the CVD risk factors, CRF measures and PA levels in SA medical students at the University of the Witwatersrand.

## Methods

### Study Design

A descriptive, cross-sectional cohort study design was used.

### Participants

Fifth-year medical students in the Graduate Entry Medical Programme (GEMP) at the Faculty of Health Sciences, University of the Witwatersrand, during a practical teaching session that involved measurement of CVD risk factors and CRF, were informed that their data may be used for future research analysis and invited to participate. Students who did not attend the clinical session or did not want their data to be used, did not have their data recorded (n = 7/140).

### Sample

Convenience sampling was used in this study.

The inclusion criteria of the study were students who participated in a teaching and learning activity as part of their curriculum. The learning activity involved measurement of CVD risk factors and CRF. Only data of students that consented were included. The data collected during this activity were retrospectively analysed.

Students who were diagnosed with CVD such as previous myocardial infarction, stroke or other forms of heart disease (such as heart failure, heart transplantation, congenital heart disease, abnormal heart rhythm or pacemaker/implantable cardiac defibrillator) were excluded from the study.

Some students did not want certain variables measured and some did not completely fill out questionnaires. Students were included in the descriptive analysis though, even if they did not have a complete data set. The reported total numbers (n) for each variable reflect this inclusion. We included the measurements we had from all students even if not a complete set of data, because measurements were mutually independent. However, only complete sets of data were used for the comparative group analysis.

Supplementary material is securely stored by the researchers and may be accessed on request, which would be considered following ethical and research integrity principles.

### Physical Activity Vital Sign

The PAVS was determined using a questionnaire (Supplemental Appendix 1). The questions were the same as those in the standard Exercise is Medicine™, Physical Activity Vital Sign.^14^ This measure quantified self-reported PA level using moderate- to vigorous-intensity activity minutes, per week.

### Pre-Participation Health Screening Questionnaire

Pre-participation screening was performed using a questionnaire based on the updated Screening Recommendations of the American College of Sports Medicine^15^ (Supplemental Appendix 2). The questionnaire also exposed the medical students to the use of a tool for assessing pre-exercise participation safety.

### Cardiovascular Disease Risk Factors

A questionnaire (Supplemental Appendix 3) was used to self-report personal CVD risk factors. The American College of Sports Medicine’s diagnostic criteria for CVD risk factors were used in the questionnaire.^16^ The criteria were used in totality and were not modified.

### Anthropometric Measures

#### Weight and Height

Weight (kg) and height (m) were measured using a Seca scale and stadiometer (Model 220, Vogel and Halke, Germany). The measurements were taken by trained, exercise physiology honours students. Weight and height were recorded twice and if the values differed by greater than 5%, a third measurement was taken. The average of the 2 closest measurements was used in the analysis.

#### Waist Circumference

Waist circumference (cm) was measured at the greatest girth between the iliac crests and the bottom of the rib cage using a standard, cloth tape measure.

#### Blood Pressure

Systolic and diastolic blood pressures (mmHg) were measured using an automated blood pressure cuff (Fora Active Plus P30, FaraCare Suisse, Switzerland). All measurements were done twice, in the seated position on the dominant arm, at heart level. The average of the 2 measurements was recorded.

#### Blood Total Cholesterol and Glucose Levels

The Accutrend® Plus (Roche, Mannheim, Germany) was used to measure total cholesterol and random blood glucose levels, as this was part of a practical teaching session and it was not possible to control for eating or determine the time of the practical sessions. Finger-prick blood samples were placed on the Accutrend cholesterol and glucose strips (Roche, Mannheim, Germany) and then read with the Accutrend machine.

### Cardio-Respiratory Fitness Test

The previously validated Watt-bike® sub-maximal ramp test^17^ (Supplemental Appendix 4) was used to predict the peak oxygen consumption.

## Statistical Analysis

Descriptive and frequency statistics were used to report the CVD risk factors, CRF measures and PA levels. Data were tested for normality using the Shapiro–Wilk test.

Statistica (v13.5) was used for all analyses.

T-tests were used to compare the normally distributed, continuous variables of students who met the World Health organization (WHO) requirements to those of students who did not meet the WHO requirements and students with low CRF vs high CRF. Mann–Whitney U tests were used for non-parametric analysis. Statistical significance was established at *P* ≤ .05.

## Ethical Considerations

Ethical clearance was obtained from the Human Research Ethics Committee of the University of the Witwatersrand (Clearance number: M200349). All measurements were explained to students at the beginning of the session, and informed consent was requested and signed by the students. Anonymity and confidentiality were preserved in that the data sheets used a study number (and no name) for each student and was kept separate from student name. Only the researchers had access to the original spreadsheet linking student name and study number. Data analysis was done using pooled data and did not identify individuals.

## Results

### Study Participant Characteristics

One hundred and thirty three students participated in the study, of which 34 (25.6%) and 90 (67.7%) were male and female, respectively (Table 1). The results indicated that this cohort of students had physical and cardio-metabolic values in the healthy ranges, except for CRF (VO_2 peak_) which was below reference ranges for the median age-group (Table 1).

**Table 1.** Physical and cardio-metabolic characteristics of a cohort of fifth-year medical students in South Africa.

Variables	N	Value (median[IR])
***Age (years)***	127	22 [2]
***Females (%)***	90	67.7
***Males (%)***	34	25.6
***Weight (kg)***	114	62 [19.5]
***Height (m)***	115	1.65 [.12]
***BMI (kg.m*** ^***−2***^ ***)***	114	22.9 [6.4]
***Waist circumference (cm)***	108	74.8 [16]
***Total cholesterol (mmol.L*** ^***−1***^ ***)***	112	3.90 [.29]
***Diastolic blood pressure (mmHg)***	115	77 [16]
*Variables*	N	*Value (mean ± SD)*
***Systolic blood pressure (mmHg)***	115	119 ± 12.5
***Random glucose (mmol.L*** ^***−1***^ ***)***	106	4.98 ± 1.01
***VO***_***2***_ ***peak (ml.min*** ^***−1***^ ***.kg*** ^***−1***^ ***)***	41	29.1 ± 5.9
***VO***_***2***_ ***peak (ml.min*** ^***−1***^ ***.kg*** ^***−1***^ ***) (Males)***	28	31.4 ± 5.6^a^
***VO***_***2***_ ***peak (ml.min*** ^***−1***^ ***.kg*** ^***−1***^ ***) (Females)***	13	27.6 ± 5.2^a^

Subjects were included if they did not have a complete data set. The reported total numbers (n) for each variable reflect this inclusion.

Abbreviations: SD, standard deviation; IR, interquartile range; VO2 peak, peak oxygen uptake.

a outside of reference ranges.^18^Reference values for age-group (20–29 years): Men: 44 mlO_2_.kg^−1^.min^−1^ and Women: 31.6 mlO_2_.kg^−1^.min^−1^

The presence of CVD risk factors features in Table 2. The highest percentages reported for CVD risk factors were for the risk factors of physical inactivity, n = 26 (19.5%) and high random glucose levels, n = 25 (18.8%) (Table 2).

**Table 2.** Cardiovascular disease risk factors^*^ of a cohort of fifth-year medical students in South Africa.

Cardiovascular Disease (CVD) Risk Factor^16^	N	n (%) of Students Presenting with the CVD Risk Factor
Age	106	0
Family History	103	11 (8.3)
Hypertension	105	10 (7.5)
Physical inactivity	104	26 (19.5)
High random blood glucose levels (≥ 7.8 mmol/l)	107	25 (18.8)
Dyslipidemia	106	6 (4.5)
Obesity	105	10 (7.5)
Smoking	103	10 (7.5)

Subjects were included if they did not have a complete data set. The reported total numbers (n) for each CVD risk factor reflect this inclusion.

* The following defining criteria were used to identify the CVD risk factors^16^: Age: Men ≥ 45 yr; women ≥ 55 yr; Family history: Myocardial infarction, coronary revascularization or sudden death before 55 yr in father or other male first-degree relative or before 65 yr in mother or other female first-degree relative; Smoking: Current cigarette smoker or those who quit within the previous 6 months or exposure to environmental tobacco smoke; Physical inactivity: Not meeting the minimum threshold of 500–1000 MET-min of moderate-to-vigorous physical activity or 75–150 min ∙ wk−1 of moderate- to vigorous-intensity physical activity and 2 days/wk of muscle-strengthening exercise; Obesity: Body mass index ≥ 30 kg ∙ m^−2^ or waist girth > 102 cm for men and > 88 cm for women; Hypertension: Systolic blood pressure ≥ 130 mmHg and/or diastolic ≥ 80 mmHg or on antihypertensive medication; Dyslipidemia: Total serum cholesterol ≥ 5.18 mmoL ∙ L^−1^

**Figure 1.**
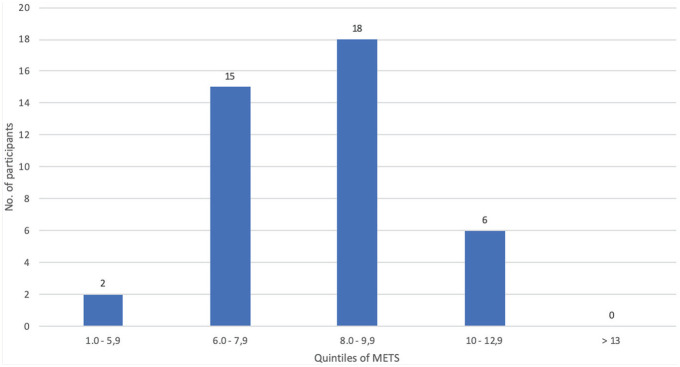
CRF, cardio-respiratory fitness, METS, metabolic equivalents

Forty-one participants (31%) agreed to and completed the CRF test. The majority of these students achieved a peak level in the range of 6.0–9.9 METS (n = 33, 80.5%) (Figure 1). Only 6 students (14.6%) achieved the optimal level of 10 – 12.9 METS and no students attained the highest quintile of ≥ 13 METS (Figure 1).

**Figure 2.**
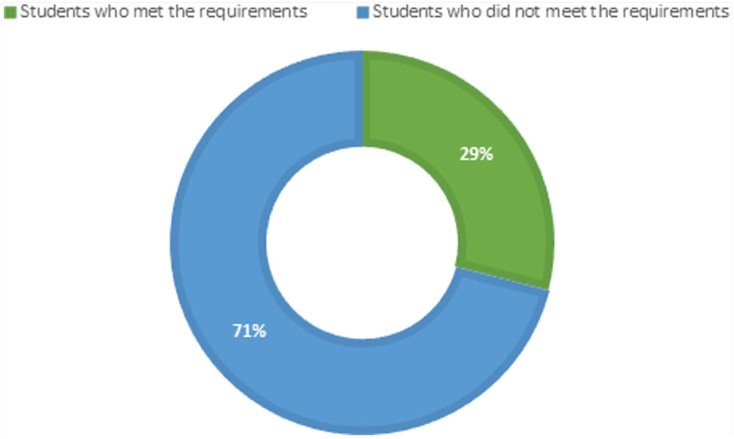
Percentage of students meeting the WHO Physical Activity requirements

One hundred and seven (107) of the students fully completed the PA questionnaire determining whether WHO PA recommendations were met. Seventy six (76) medical students (71%) reported not meeting the WHO’s PA dose recommendations (Figure 2).

T-tests and Mann–Whitney U tests showed no significant differences in cardio-metabolic parameters between students that met the WHO, PA requirements and those that did not meet the WHO requirements (Table 3).

**Table 3.** Comparison of fifth-year medical students who are meeting World Health Organization (WHO) physical activity requirements* to those who are not meeting the requirements.

Variables	Students Not Meeting the WHO requirements^a^ (n = 41)	Students Meeting the WHO requirements^a^ (n = 18)	p-value
***Weight (kg)***	61 [17.9]	64 [38.7]	.99^c^
***BMI (kg.m*** ^***-2***^ ***)***	23.8 [6.2]	23.9 [7.9]	.44^c^
***Waist circumference (cm)***	75 [10]	76 [24]	.94^c^
***Total cholesterol (mmol.L*** ^***−1***^ ***)***	3.95 [.49]	3.90 [.21]	.20^c^
***Systolic blood pressure (mmHg)***	118 ± 11.7	121 ± 16.3	.56^b^
***Diastolic blood pressure (mmHg)***	74 [14]	73 [15]	.75^c^
***Random glucose (mmol.L*** ^***−1***^ ***)***	4.79 ± 1.07	4.80 ± .95	.99^b^
***VO***_***2***_ ***peak (ml.min*** ^***−1***^ ***.kg*** ^***−1***^ ***)***	28.3 ± 5.5	29.9 ± 6.6	.50^b^

Data depicted as mean ± SD or median [interquartile range].

Abbreviations: BMI, body mass index; VO2 peak = peak oxygen uptake.

a Adults should do at least 150–300 min of moderate-intensity aerobic physical activity, or at least 75–150 min of vigorous-intensity aerobic physical activity, or an equivalent combination of moderate-intensity and vigorous-intensity activity throughout the week and should also do muscle-strengthening activities at moderate or greater intensity that involve all major muscle groups on 2 or more days a week.^19^

b T-test *P*-value (normally distributed data).

c Mann–Whitney U test, *P*-value (for skewed data sets).

However, there were significant differences between students who had high CRF (i.e. achieved ≥ 8 METS for the VO_2_ peak test), and those with low cardio-respiratory fitness (i.e. achieved < 8 METS for the VO_2_ peak test) (Table 4). Students with higher CRF levels had lower weight (*P* = .003), lower BMI (*P* = .0005) and lower waist circumference (*P* = .02).

**Table 4.** Comparison of fifth-year medical students with low cardio-respiratory levels to those with high cardio-respiratory levels.

Variables	Students with Low CRF (i.e. < 8 METS) (n = 17)	Students with High CRF (i.e. ≥ 8 METS) (n = 24)	*P*-value
***Weight (kg)***	68.1 [11.7]	58.4 [15.3]	**.003** ^b^
***BMI (kg.m*** ^***-2***^ ***)***	26.4 [3.8]	21.9 [4.3]	**.0005** ^b^
***Waist circumference (cm)***	79 [9]	73 [10]	**.02** ^b^
***Total cholesterol (mmol.L*** ^***−1***^ ***)***	3.94 [.61]	3.90 [.21]	.27^b^
***Systolic blood pressure (mmHg)***	123 ± 11.6	118 ± 12.6	.30^a^
***Diastolic blood pressure (mmHg)***	79 [15]	77 [11]	.39^b^
***Random glucose (mmol.L*** ^***−1***^ ***)***	4.97 ± .91	4.54 ± 1.09	.21^a^

CRF = cardio-respiratory fitness.

**Bolded** = *P*-value <.05.

Data depicted as mean ± SD or median [interquartile range].

a T-test *P*-value (normally distributed data).

b Mann–Whitney U test, *P*-value (for skewed data sets).

## Discussion

This cohort of medical students was generally in the healthy, normal ranges with respect to body mass index, waist circumference, blood pressures, blood total cholesterol and glucose levels (Table 1). There is a lack of research of CRF measures for medical students in South Africa and, to the authors’ knowledge, this study is the first. The VO_2__peak_ (CRF) values were in the low fitness, age-related category according to the normal ranges set out by the FRIEND study.^18^ Physical inactivity and high random glucose levels are reported as the highest attributable fraction to the CVD risk factors among this cohort of medical students (Table 2). In addition, a large proportion of the medical students did not meet the PA levels recommended by the (WHO)^19^ (Figure 2).

Previously, Nyombi et al (2016)^20^ described CVD risk factors of medical students at a university in Africa (Uganda). In contrast to our study, Nyombi et al (2016)^20^ found alcohol consumption (31.7%), elevated systolic blood pressure (14%) and excessive salt intake (13%) to be the CVD risk factors with the highest prevalence, whereas our study found physical inactivity (19.5%) as the CVD risk factor with the highest prevalence (Table 2). It must be noted, however, that the questionnaire used by Nyombi et al (2016)^20^ did not include any questions about PA habits or levels. Similarly, another study describing CVD risk factors of university students in Africa did not investigate PA levels but found that obesity and dyslipidemia had a high prevalence among university students in Ghana.^21^

Studies of medical students on other continents have found obesity and hypertension,^22,23^ physical inactivity, high-fat diet^22,24^ and dyslipidemia^25^ to be highly prevalent as CVD risk factors. The different methodologies used to measure CVD risk factors must be considered in explaining the disparity in results among the cited studies.

A sub-maximal, graded exercise test was used in this study to determine CRF level by estimating VO_2__peak_ with the mean peak VO_2_ values for males and females falling in the lower 10^th^ percentile of the age and gender reference standards for CRF^18^ (Table 1) and are lower than those reported for young South African adults by Prioreschi et al (2017)^26^ (males: median[IQ] 41.9(41, 43) and females: 32.6(32, 33) mlO_2_.min^−1^.kg^−1^.

Furthermore, a maximal level of ≥ 8 METS attained during a CRF test has been associated with low mortality rates.^27^ Only 24 (58%) of the students achieved this protective value during the CRF test and 6 (14%) attained a level of ≥ 10 METS (moderately fit level) (Figure 1). Thus, there is a health-related need to increase CRF levels in South African medical students.

A study of students at a Kenyan University^28^ also reported ‘VO_2max_ well below the expected average’. In addition, Nabi et al (2015)^29^ found below average (fair) VO_2max_ scores for medical students at a university in India, suggesting compromised CRF among university students.

A reason for low CRF levels may be the high physical inactivity levels (Table 2) and low PA levels (Figure 2) reported by the medical students. This however, was not the case when comparing the students who met the WHO, PA requirements to those that were not meeting the requirements (Table 3). Nabi et al (2015)^29^ though noted that low CRF levels could be attributed to the decreased PA and to the unhealthy lifestyle behaviours that are established during the years of medical school education.

Although VO_2_ peak was not significantly different between students that met and did not meet the WHO, PA requirements (Table 3), a comparison of the medical students with low CRF vs high CRF showed that VO_2_ peak was associated with weight, BMI and waist circumference. Previous research has also found an association between CRF and weight, BMI and waist circumference.^30^ This may highlight the importance of targeting CRF in medical students as a weight and visceral fat management tool.

Seventy one percent of the medical students in this study reported being physically inactive (Figure 2), and only 29% of the medical students met the WHO, PA requirements. This finding is in contrast to the research with medical students in Canada (64% met the WHO, PA recommendations)^31^ and the USA (61% met the CDC, PA recommendation).^32^ The South African medical students of this study were physically inactive compared to their North American counterparts.

This raises the question that if our future medical doctors are inactive, will they promote PA whilst counselling patients? Research has noted that the personal health and lifestyle practices of future medical doctors influence their counselling activities.^12,13^ Raza et al (2010)^24^ found that if a medical student is physically active, their propensity for PA counselling is increased. This may be a substantial reason for promoting interventions that increase PA among medical students in South Africa.

Students studying towards a medical degree can have numerous challenges which may contribute to the adoption of a sedentary lifestyle. High academic demands, a lack of time and exercise and irregular diets are some factors highlighted by research.^33^ These barriers need to be addressed when interventions are implemented.

A limitation of this study is that students sometimes chose not to record or do some of the tests; thus, we were not able to get full sets of testing data for all 133 participants. In addition, blood tests were finger-prick tests and performed under non-fasting conditions and PA levels were self-reported. Future research should evaluate the PA levels of medical students using objective measures (e.g. accelerometer).

## Conclusion

The most significant finding of this study was that fifth-year medical students at a South African university were not meeting the PA levels recommended to maintain health and lower CVD risk, and the students recorded low levels of CRF. Medical students are the future custodians of physical activity promotion and counselling as a tool for the treatment and prevention of chronic diseases/diseases of lifestyle. If medical students do not lead a physically active lifestyle themselves, the probability of encouraging their future patients to do so is likely to be significantly reduced. Intervention programmes that increase the PA and CRF levels of medical students need to be designed and implemented in order to protect the health of future medical doctors and promote the use of PA as a medicine.

## Supplemental Material

sj-pdf-1-ajl-10.1177_15598276221089888 – Supplemental material for Cardio-Respiratory Fitness and Cardiovascular Disease Risk Factors Among South African Medical Students Supplemental material, sj-pdf-1-ajl-10.1177_15598276221089888 for Cardio-Respiratory Fitness and Cardiovascular Disease Risk Factors Among South African Medical Students by Georgia Torres, Neil F Gordon and Demitri Constantinou in American Journal of Lifestyle Medicine
