# Supplementary material for: Cardio-Respiratory Fitness and Cardiovascular Disease Risk Factors Among South African Medical Students
Source: Am J Lifestyle Med. 2022 May 23;17(6):791–8. doi: 10.1177/15598276221089888 (PMC10948929; doi:10.1177/15598276221089888)
Supplement: sj-pdf-1-ajl-10.1177_15598276221089888 – Supplemental material for Cardio-Respiratory Fitness and Cardiovascular Disease Risk Factors Among South African Medical Students [file sj-pdf-1-ajl-10.1177_15598276221089888.pdf]

1: PHYSICAL ACTIVITY VITAL SIGN QUESTIONNAIRE

(Extracted from the www.exerciseismedicine.org)

Physical Activity Vital Sign

Exercise is Medicine® | AMERICAN COLLEGE of SPORTS MEDICINE®

Study Number:

1. On average, how many days per week do you engage in moderate to strenuous exercise (like a brisk walk)?	_____	days
2. On average, how many minutes do you engage in exercise at this level?	_____	minutes
**Total minutes per week of physical activity (multiply #1 by #2)**	_____	**minutes per week**

Incorporate the PAVS into your electronic health record and patient intake forms. Calculations may be programmed and the sedentary patient flagged for referral or counseling.

Using the Physical Activity Vital Sign

National guidelines recommend 150 minutes per week of moderate intensity physical activity. That's just $2\,{}^{1}/_{2}$ hours out of 168 hours in a week! In place of moderate intensity activity, you can also complete 75 minutes of vigorous intensity physical activity, or an equivalent combination of moderate and vigorous intensity physical activity.

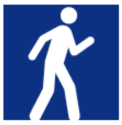

What's Moderate Intensity?

- You can talk, but not sing, while performing the activity.
- Examples: brisk walking, slow biking, doubles tennis, various forms of dance, active home chores and gardening, etc.

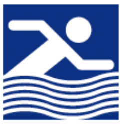

What's Vigorous Intensity?

- Vigorous intensity: You can no longer talk easily during the activity and are somewhat out of breath.
- Examples: jogging, fast bicycling, singles tennis, aerobic exercise class, swimming laps, etc.

You can also achieve 150 minutes through a combination of moderate and vigorous intensity physical activity.

- 1 minute of vigorous activity is equal to 2 minutes of moderate activity.
- If activity is done throughout the day, you can perform multiple "bouts" of any length to add up to the recommended 150 minutes/week.

If your patient is NOT achieving 150 minutes a week of physical activity, help the patient to set more realistic goals to gradually increase either their frequency or duration until they are capable of safely achieving the national recommendations.

The Physical Activity Vital Sign – Other Considerations

- A comprehensive assessment of physical activity should include promotion of active living throughout the day to reduce sedentary time/screen time, as well as muscle strengthening exercises as recommended by the Physical Activity Guidelines for Americans: Adults should do muscle strengthening activities that are moderate or high intensity and involve all major muscle groups on 2 or more days a week.
- If you wish to add a question on muscle strengthening activities, we recommend the following:

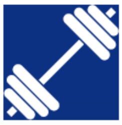

How many days a week do you perform muscle strengthening exercises, such as bodyweight exercises or resistance training?	_____	days

APPENDIX 2: Pre-participation Health Screening questionnaire

PRE-PARTICIPATION HEALTH SCREENING QUESTIONNAIRE
STUDY NUMBER:………………..

SECTION A

Signs and Symptoms:

Please select any of the signs or symptoms that you have recently experienced:

- Pain, discomfort in the chest, neck, jaw or arms at rest or upon exertion
- Shortness of breath at rest or with mild exertion
- Dizziness or loss of consciousness during or shortly after exercise
- Shortness of breath occurring at rest or 2 – 5 hours after the onset of sleep
- Edema (swelling) in both ankles that is most evident at night or swelling in a limb
- An unpleasant awareness of forceful or rapid beating of the heart
- Pain in the legs or elsewhere while walking; often more severe when walking upstairs/uphill
- Known heart murmur
- Unusual fatigue or shortness of breath with usual activities

☐ Yes
☐ No

SECTION B

Current Physical Activity
When answering the questions, please note the following:
Moderate intensity: An activity that causes noticeable increases in heart rate and breathing (eg. Brisk walking)
Vigorous intensity: An activity that causes substantial increases in heat rate and breathing (eg. Jogging)

Over the last three months, have you regularly participated in physical activity for at least 30 minutes, three days/week at moderate intensity?

☐ Yes
☐ No

If Yes, which of the following best describes any vigorous intensity activity in your regular routine in the last three months?

- I participate in some or all vigorous intensity activity
- None, but I want to begin some vigorous intensity activity

- None, and I want to continue moderate intensity activity only

<u>SECTION C</u>

<u>Medical conditions (Part 1)</u>

Please select any of the following medical conditions that you currently have or have had:

- Heart attack
- Heart surgery
- Cardiac catheterization
- Coronary angioplasty (PTCA)
- Heart valve disease
- Heart failure
- Heart transplantation
- Congenital heart disease
- Abnormal heart rhythm
- Pacemaker/implantable cardiac defibrillator
- Peripheral vascular disease – disease affecting blood vessels in arms, hands, legs and feet
- Cerebrovascular disease – Stroke or transient ischemic attack
- Type 1 or Type 2 diabetes
- Renal (kidney) disease

<u>Medical conditions (Part 2)</u>

Please select any of the following conditions that you currently have or have had:

- Cancer
- Abnormal Blood Lipid levels (Dyslipidaemia)
- High blood pressure (Hypertension)
- Pre-diabetes (Metabolic Syndrome)
- Pulmonary Disease
- Depression

<u>Name: ______________________________ Date:</u>

<u>Signature:</u>

APPENDIX 3: Self-reported cardiovascular disease risk factors table

Study number:__________Date:____________________

Please tick as applicable

Cardiovascular Disease Risk Factors Questionnaire

DO YOU TAKE PRESCRIPTION MEDICATIONS? LIST BELOW:

Cardiovascular Risk Factors:

☐ You are a man older than 45 years	☐ You are a woman older than 55 years, you have had a hysterectomy, or you are post-menopausal
☐ You smoke or quit within the previous 6 months or high exposure	☐ Your blood cholesterol level is ≥ 5.2 mmol/L or LDL ≥ 3.37 mmol/L or HDL ≤ 1.04 mmol/L or on lipid-lowering medications
☐ Your BP is ≥140/90 or on anti-hypertensive medications	☐ You don't know your cholesterol level
☐ You don't know your BP	☐ You have a close blood relative who had a heart attack, cardiac revascularization before age 55 (father/brother), or age 65 (mother/sister)
☐ You are physically inactive (Less than 30 min of moderate intensity activity, at least 3 days per week, for the last 3 months)	☐ BMI ≥ 30kg.m^2 ; Waist circumference ≥102cm for males ≥ 88cm for women
☐ Fasting blood glucose levels ≥ 5.6 and < 6.94 mmol/L (OGTT: ≥ 7.8 and ≤ 11 mmol/L)	☐ HDL ≥ 1.6 mmol/L (Negative Risk Factor)

APPENDIX 4: WATT-BIKE, SUB-MAXIMAL RAMP TEST PROCEDURE

What is the submaximal ramp test

During this test you will ride in 15 watt increments until you experience a perceived rate of exertion (RPE) of 7 out of 10 (where 1= easy, 10= extremely hard), you'll no longer be able to maintain a conversation when you reach an RPE of 7.

How to complete the submaximal ramp test

The Model B Performance Monitor has a pre-programmed submax ramp test. Simply follow the instructions below to get started:

Set your air resistance to level 8 on the Wattbike Trainer or 3 on the Wattbike Pro.

1. From the main menu select workouts/ tests

2. On the test screen, select Submax Ramp

3. Input your age, weight in kg's and gender

4. Input your starting Watts (we recommend 55W if you're unfit or 100W if you're regularly exercising) then press enter

5. The live display will show your current interval and target watts

6. Once you have reached 7/10 on the Borg scale of exertion, press escape to end the test.
